# When ultra-processing obscures sustainable dietary transitions

**DOI:** 10.3389/fnut.2026.1908823

**Published:** 2026-08-19

**Authors:** Miguel López-Moreno, José Francisco López-Gil

**Affiliations:** 1 Diet, Planetary Health and Performance, Faculty of Health Sciences, Universidad Francisco de Vitoria, Pozuelo, Spain; 2 Institute of Health and Sport Sciences, Faculty of Health Sciences, Universidad Francisco de Vitoria, Madrid, Spain; 3 School of Medicine, Universidad Espíritu Santo, Samborondón, Ecuador; 4 Vicerrectoría de Investigación y Postgrado, Universidad de Los Lagos, Osorno, Chile

**Keywords:** food processing, NOVA classification, nutritional epidemiology, sustainable diets, ultraprocessed food

## Introduction

Ultra-processed foods (UPFs) have been increasingly associated with adverse cardiometabolic outcomes and higher all-cause mortality across observational and experimental research (1–3). However, the growing use of UPFs as a single-category exposure in scientific communication and public health messaging may overlook an increasingly evident reality. Foods classified within the same NOVA classification of food processing (NOVA) category often differ substantially in nutritional composition, metabolic effects, and clinical associations (4).

## The heterogeneity of ultra-processed foods

Recent evidence suggests that the health associations of UPFs are highly heterogeneous (5). In several prospective cohorts, processed meats and sugar-sweetened beverages consistently showed positive associations with type 2 diabetes and cardiometabolic disease, whereas other UPF subgroups, including breads or breakfast cereals, showed neutral or inverse associations (6–8). In the European Prospective Investigation into Cancer and Nutrition (EPIC) cohort, higher consumption of ultra-processed animal-based products and sweetened beverages was associated with greater diabetes risk, while ultra-processed breads, cereals, and plant-based alternatives were associated with lower risk estimates (9). This does not invalidate the conceptual basis of NOVA, which captures industrial processing rather than nutritional quality. However, aggregating biologically diverse foods into a single exposure category may obscure etiologically relevant heterogeneity and complicate causal interpretation by treating heterogeneous dietary exposures as biologically equivalent interventions (10).

Beyond heterogeneity in nutrient composition, interpreting UPFs as a single exposure may also create challenges for causal inference. In nutritional epidemiology, foods are rarely consumed in isolation; increasing the intake of one food generally implies reducing another. Consequently, the health effects attributed to a UPF category may depend not only on the food itself but also on the dietary alternative it replaces (11). Grouping nutritionally distinct foods into a common category may therefore obscure meaningful differences in substitution effects and lead to oversimplified interpretations of dietary risk (12). From a counterfactual perspective, the relevant question is often not whether a food is ultra-processed, but whether it represents an improvement or deterioration relative to the realistic alternative available to consumers (10).

These findings do not invalidate concerns regarding many UPFs. Rather, they highlight the limitations of treating ultra-processing itself as a nutritionally coherent exposure independent of food matrix, nutrient composition, or replacement context. This does not exclude the possibility that certain non-nutritional features of ultra-processing, including additives, emulsifiers, or altered food matrices, may contribute more uniformly to disease risk across some UPF subgroups. This may be particularly relevant in the context of sustainable dietary transitions (13, 14). Certain plant-based alternatives are increasingly grouped within the same risk narrative as processed meats and confectionery despite markedly different nutritional characteristics. Although some products remain high in sodium or additives (15, 16), substitution trials suggest that replacing animal products with selected plant-based alternatives may modestly improve low-density lipoprotein cholesterol (LDL-C) and body weight (17).

## Ultra-processing, substitution, and sustainable dietary transitions

**Figure 1:**
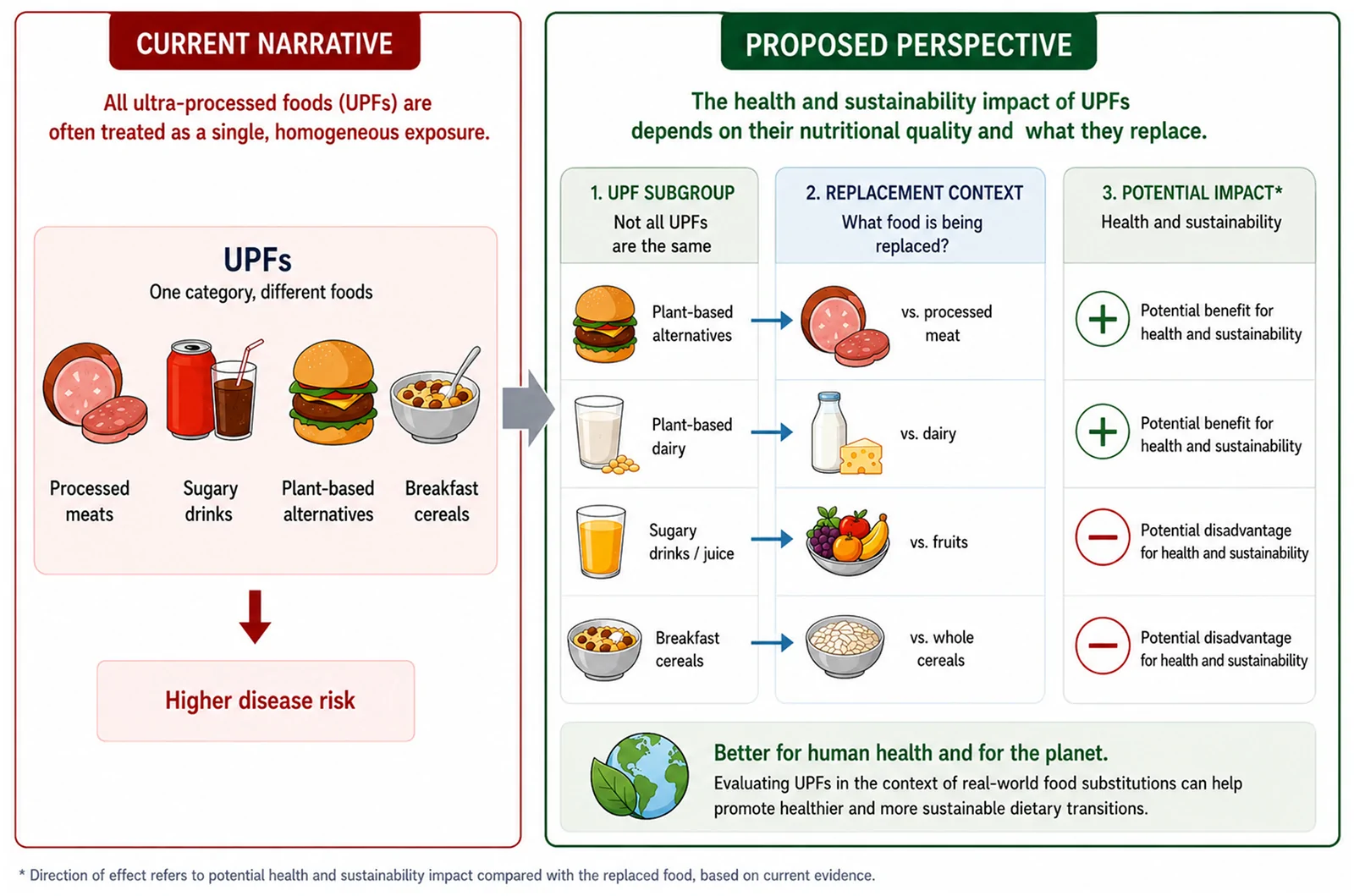
Reframing the health and sustainability implications of ultra-processed foods according to food subgroup and replacement context.

The growing interest in sustainable dietary patterns highlights the importance of considering food substitutions rather than food categories in isolation. Current dietary guidelines increasingly encourage a greater consumption of plant-derived foods to improve both human and planetary health (18, 19). However, many products that facilitate this transition, including fortified plant-based beverages, meat alternatives, and other convenience foods, are frequently classified as UPFs (20). As a result, consumers may receive conflicting messages: one recommending a shift toward more plant-based diets and another discouraging many of the products that can support such a transition (Figure 1).

This tension does not imply that all plant-based alternatives should be considered healthy by default. Their nutritional quality varies considerably and some products contain excessive amounts of sodium, refined starches, or additives (21). Nevertheless, evaluating these foods exclusively through the lens of processing may overlook their potential role as replacements for foods with less favorable health or environmental profiles (5). This consideration may be particularly relevant given that several dietary models proposed to improve environmental sustainability rely on increased consumption of plant-based foods, some of which are currently classified as ultra-processed (22). In practice, the relevant comparison may not be between a plant-based burger and minimally processed legumes, but between a plant-based burger and the processed meat product it is replacing.

## Discussion

Public health nutrition should continue discouraging dietary patterns dominated by hyperpalatable foods rich in added sugars, sodium, and saturated fats. However, framing all UPFs as a nutritionally homogeneous category may obscure important differences in health effects, mechanisms, and practical dietary applications. While the NOVA classification provides a useful framework for studying industrial food processing, it was not designed to evaluate overall nutritional quality or to guide food substitutions in clinical practice.

Furthermore, an additional methodological challenge deserves consideration. Most dietary assessment instruments used in large epidemiological studies, particularly food frequency questionnaires (FFQs), were originally designed to estimate habitual food intake rather than to characterize the degree of industrial processing. Consequently, information on product formulation, ingredient lists, reformulation over time, and the presence of specific additives is often unavailable or insufficient to accurately classify foods according to NOVA or similar processing-based systems. This requires retrospective food classification and may lead to exposure misclassification, potentially attenuating or distorting observed associations between UPF consumption and health outcomes (23, 24). Moreover, both the agreement between food processing classification systems and the validity of currently available instruments for assessing UPF intake are only moderate, further highlighting the uncertainty surrounding current approaches to quantifying UPF exposure (24, 25). These methodological limitations should be considered when interpreting the growing body of observational evidence linking UPFs with health outcomes.

Future research should move beyond aggregate UPF measures and improve dietary assessment methods capable of more accurately capturing food processing characteristics, including changes in product formulation and the presence of relevant additives. Investigating specific food subgroups, realistic dietary replacement scenarios, and underlying biological mechanisms may help distinguish the effects attributable to processing itself from those related to nutrient composition, food matrix characteristics, and broader dietary patterns. A more nuanced interpretation of ultra-processing may improve both scientific understanding and public health communication while avoiding unintended barriers to healthier and more sustainable dietary transitions. Ultimately, public health recommendations may benefit more from identifying which foods should replace others within real-world dietary patterns than from relying exclusively on the degree of industrial processing as a marker of healthfulness.
